# Radiation Exposure Reduction and Patient Outcome by Using Very Low Frame Rate Fluoroscopy Protocol (3.8 + 7.5 fps) During Percutaneous Coronary Intervention

**DOI:** 10.3389/fcvm.2021.625873

**Published:** 2021-02-09

**Authors:** Ankush Gupta, Sanya Chhikara, Rajesh Vijayvergiya, Parag Barwad, Krishna Prasad, Rajat Datta, Nalin K. Mahesh, Preetika Maurya, Navreet Singh

**Affiliations:** ^1^Department of Cardiology, Military Hospital Jaipur, Jaipur, India; ^2^Vardhman Mahavir Medical College, & Safdarjung Hospital, New Delhi, India; ^3^Department of Cardiology, Advanced Cardiac Centre, Post Graduate Institute of Medical Education and Research Chandigarh, Chandigarh, India; ^4^Department of Cardiology, Army Hospital Research & Referral, New Delhi, India; ^5^Department of Cardiology, Apollo Adlux Hospital, Kochi, India; ^6^Base Hospital Delhi Cantt, New Delhi, India; ^7^Department of Cardiology, The Air Force Central Medical Establishment, New Delhi, India

**Keywords:** percutaneous coronary intervention, fluoroscopy, air kerma, radiation, frame rate, cine acquisition

## Abstract

**Objectives:** In this study, we intend to analyze the feasibility and efficacy of very low frame rate fluoroscopy (VLFF) protocol using a combination of 3.8 and 7.5 fps while performing Percutaneous Coronary Intervention (PCI).

**Methods:** A retrospective cohort including 193 patients undergoing PCI under the VLFF protocol (Post-VLFF group) was compared with a retrospective cohort of 133 patients, who underwent PCI prior to implementation of VLFF protocol (Pre-VLFF group). In the Pre-VLFF group, all PCIs were performed using fluoroscopy frame rate of 15 fps. In the Post-VLFF group, 3.8 fps was used to guide catheter engagement, coronary lesion wiring, pre-and post-dilation, and 7.5 fps was used for lesion assessment and stent placement. Increasing use of fluoroscopic storage in place of cineangiography was also encouraged. Cine acquisition in both groups was performed at 15 fps. Primary endpoint was radiation exposure measured by Air Kerma. Secondary endpoints were procedure related outcomes and patient related outcomes (Major Adverse Cardiac Events including all-cause mortality, Target Lesion Failure, Myocardial Infarction, and Stroke).

**RESULTS:** Post-VLFF group showed 74.7% reduction in Air Kerma as compared to Pre-VLFF group (433 ± 27 mGy vs. 1,714 ± 140 mGy; *p* < 0.0001), with no increase in the fluoroscopy time (15.38 ± 0.98 min Post-VLFF vs. 17.06 ± 1.29 min Pre-VLFF; *p* = 0.529) and contrast volume (116.5 ± 4.9 ml Post-VLFF vs. 116.7 ± 6 ml Pre-VLFF; *p* = 0.700). Both groups had comparable procedural success and complications rates as well as incidence of MACE.

**Conclusions:** The very low frame rate fluoroscopy protocol is a feasible, effective, and safe method to significantly reduce the radiation exposure during PCI without any compromise on procedural and patient outcomes.

## Introduction

Percutaneous coronary intervention (PCI) has an indispensable role in the management of obstructive coronary artery disease. What started with the advent of balloon angioplasty in 1977 by Andreas Gruntzig has now progressed to complex interventional procedures and implantation of drug eluting stents (1).

The constant advancement of interventional cardiology has enabled us to attempt increasingly complex procedures which inadvertently has led to increase in fluoroscopy time and radiation exposure. Thus, it is imperative to monitor and minimize the hazardous effects of ionizing radiation to both the patient and health care providers (2).

The side effects of radiation exposure include cancer, cataracts, non-malignant skin damage, and impaired fertility (3). Several studies have confirmed the increased incidence of cancer as well as posterior subcapsular lens opacities in interventional cardiologists (4). There are also concerns over the possibility of a causal relationship between left sided brain tumors and occupational radiation exposure in interventional cardiologists (5).

Thus, a variety of strategies are being utilized for reducing radiation exposure in a cardiac catheterisation laboratory. In addition to the use of personal protection apparels and devices, radiation dose optimisation by using lower fluoroscopy frame rates per second (fps) and selective fluoroscopic storage instead of cine acquisition are described in the past (6–8). Decreasing fluoroscopic frame rate from 15 to 7.5 fps has been described to reduce radiation dose exposure during PCI without affecting the quality of imaging. (7, 9–11).

In this study, we propose to examine the feasibility and efficacy of a novel very Low Frame rate Fluoroscopy (VLFF) protocol during PCI and its effect on radiation exposure, procedural outcome and patient related outcomes assessed at 1 year.

## Methods

Data of a retrospective cohort of 193 consecutive patients undergoing PCI under VLFF (Post-VLFF group) protocol was compared with a retrospective cohort of 133 consecutive patients who underwent PCI prior to implementation of VLFF protocol (Pre-VLFF group). The study protocol was reviewed and approved by the hospital's ethics committee (institutional review board).

In December 2018, we adopted a new protocol for fluoroscopy while performing PCI called Very low fluoroscopy frame rate (VLFF) protocol. It consisted of: (i) use of fluoroscopy at 3.8 fps for advancement of guide catheter till aortic root, engaging the guide catheter, wiring the coronary artery, tracking the pre-dilation and post-dilation balloon; (ii) Fluoroscopy of 7.5 fps was used for lesion assessment and stent positioning; (iii) Increasing the use of fluoroscopy store instead of cine for retrospective review; (iv) Cine was used only for baseline angiography, assessing procedural complications, and final angioplasty result. We implemented the protocol across the entire spectrum of interventions including primary PCI, chronic total occlusions (CTO), Left Main intervention, rotablation, and bifurcation PCI. Prior to this, the standard protocol was to perform both fluoroscopy and Cine acquisition at 15 fps. All procedures were performed using a single plane on Philips Allura Xper FD20/10 flat plane detector biplane digital angiography system (Philips Medical systems, The Netherlands).

Data for analysis was collected form out patient charts, hospital charts and catheterization laboratory database for both group of patients. The pre-VLFF group included consecutive patients who underwent PCI between September to November 2018, while the Post-VLFF group included those who underwent PCI during January–March 2019. Interventions performed during the month of December 2018 were considered transition period for interventions, to create an optimal VLFF strategy and to provide an adjustment period for the operator and laboratory staff to acquaint themselves to the machine settings and image quality. Patient characteristics studied were age, sex, BMI, left ventricular ejection fraction (LVEF), indication of PCI, and MACE. Procedural characteristics included catheterisation route, number of vessels addressed, number of stents placed, complexity of procedure, fluoroscopy time, procedural outcome, amount of contrast used, and procedural complications. All cine acquisition runs were recorded at 15 fps in both the groups and there was no change in the catheterisation laboratory personnel or any other procedural and machine settings throughout the duration of this study.

The primary endpoint of this study was radiation exposure to the patient in the form of Air Kerma measured in milligray (mGy). Secondary outcomes were procedure related and patient related outcomes described earlier. All patients included in the study were followed up prospectively to determine patient related outcomes at 1 year. For the purpose of this study MACE was defined as a composite endpoint of all-cause mortality, Target Lesion Failure (TLF), myocardial Infarction (MI), and stroke (12).

Statistical analysis was done using IBM SPSS version 24.0. The categorical variables were expressed as percentages, the continuous variables were expressed as mean ± standard deviation. Univariate as well as multivariate analysis was performed. For univariate analysis the categorical variables were compared using the chi-square test/fisher exact test as applicable. The continuous variables were compared using the Mann-Whitney U test.

A multivariate analysis was also done to know the factors which were independently associated with radiation exposure. Multivariate analysis was performed using linear regression. For linear regression Air kerma (radiation exposure) was taken as the dependent variable, the independent variables selected were age, gender, indication for the procedure, ejection fraction, number of stents, route of angiography, fluoroscopy time and frame rate. Regression coefficients and respective 95% confidence intervals were calculated. All *p* < 0.05 were taken as significant.

## Results

Data of 370 patients who underwent PCI during the above-mentioned study period at our center was available for analysis. Out of these 44 were excluded due to loss to follow up or incomplete details in the institutional database. Finally data of 326 patients was analyzed. Of these, 133 patients were in the Pre-VLFF group and 193 in the Post-VLFF group. Baseline characteristics (Table 1) showed no significant differences between the two groups in terms of age, sex, BMI, indication of PCI, and LVEF at presentation.

**Table 1:** Baseline characteristics for all the patients (*n* = 326) as well as the Pre-VLFF (*n* = 133) and Post-VLFF (*n* = 193) subgroups.

**Baseline characteristics**	**All (*n* = 326)**	**Pre-VLFF 15 fps (*n* = 133)**	**Post-VLFF 3.8 + 7.5 fps (*n* = 193)**	***p*-value**
Age [Mean ± SD]	61 ± 10	61 ± 11	60 ± 9	0.427
Sex [*n*(%)]				0.769
Male	272 (83.4)	110 (82.7)	162 (83.9)	
Female	54 (16.6)	23 (17.3)	31 (16.1)	
BMI [Mean ± SD]	27.3 ± 7.1	27.2 ± 6.9	26.9 ± 7.3	0.731
Procedural indication [*n*(%)]				0.950
CSA	117 (35.9)	48 (36.1)	69 (35.8)	
ACS	209 (64.1)	85 (63.9)	124 (64.2)	
EF [Mean ± SD]	49 ± 10	48 ± 10	49 ± 11	0.287

*Values are n (%) or mean ± SD*.

*ACS, Acute Coronary Syndrome; BMI, Body Mass Index; CSA, Chronic Stable Angina; EF, Ejection Fraction; fps, frames per second; VLFF, Very Low Frame Rate Fluoroscopy*.

The two groups showed no difference with respect to the number of vessels addressed, number of complex procedures, number of stents placed, success rate and procedure related complications (Table 2). There was a statistically significant difference between the two groups with respect to route for intervention: femoral route (27.8% in the Pre-VLFF group vs. 15% in the post-VLFF group, *p* = 0.004), radial route (61.7% in the Pre-VLFF group vs. 74.6% in the post-VLFF group, *p* = 0.012). Both groups were identical as far as transulnar approach for PCI was concerned. There was no difference in the rate of procedural complications (0.8% in pre-VLFF group vs. 1% in Post-VLFF group; *p* = 0.792) and procedural success rate (97.7% in pre-VLFF vs. 99% in post-VLFF group; *p* = 0.379).

**Table 2:** Procedural characteristics of all patients as well as Pre-VLFF and Post-VLFF subgroups.

**Procedural characteristics**	**All (*n* = 326)**	**Pre-VLFF 15 fps (*n* = 133)**	**Post-VLFF 3.8 + 7.5 fps (*n* = 193)**	***P*-value**
**Route**				0.016
Radial	226 (69.3)	82 (61.7)	144 (74.6)	0.012
Femoral	66 (20.2)	37 (27.8)	29 (15)	0.004
Ulnar	34 (10.4)	14 (10.5)	20 (10.4)	0.962
**Number of stents**	1.49 ± 0.85	1.42 ± 0.81	1.54 ± 0.87	0.287
**Number of vessels**				
1	299 (91.7)	122 (91.7)	177 (91.7)	0.995
2	25 (7.6)	11 (7.6)	14 (7.2)	0.735
≥3	2 (0.6)	0 (0)	2 (1.0)	0.239
**Primary**	28 (8.6)	15 (11.3)	13 (6.7)	0.150
**Complex procedures**	122 (37.4)	52 (39.1)	70 (36.3)	0.604
Multivessel	27 (8.2)	11 (8.3)	16 (8.2)	0.995
LM	29 (8.9)	11 (8.3)	18 (9.3)	0.742
Bifurcation	24 (7.4)	12 (9.0)	12 (6.2)	0.341
ROTA	15 (4.6)	6 (4.5)	9 (4.7)	0.949
CTO	27 (8.3)	12 (9)	15 (7.8)	0.687
**Successful**	321 (98.5)	130 (97.7)	191 (99)	0.379
**Procedural complications**	3 (0.9)	1 (0.8)	2 (1)	0.792

*Values are n (%) or mean ± SD; CTO, Chronic Total Occlusion; fps, frames per second; VLFF, Very Low Frame Rate Fluoroscopy; LM, Left Main Artery; ROTA, rotational atherectomy*.

The fluoroscopy time was similar in the two groups (17.06 ± 1.29 min in Pre-VLFF group & 15.38 ± 0.98 min in Post-VLFF group, *p* = 0.529). Moreover, the use of lower frame rates did not lead to an increase in contrast use (Mean contrast volume 116.7 ± 6 ml in the Pre-VLFF group vs. 116.5 ± 4.9 ml in the Post-VLFF group; *p* = 0.700). There was a highly significant decrease in Air Kerma (AK) in the Post-VLFF group compared to the pre-VLFF (mean 1,714 ± 140 mGy in the pre-VLFF group vs. mean 433 ± 27 mGy in the post-VLFF group; and median (range) was 1,197(683–2,062) in the pre-VLFF group vs. 302 (206–517) in post-VLFF group; *p* < 0.0001). The new protocol led to a 74.7% reduction in mean AK. The patient related outcomes (MACE) at 1 year showed a non-significant difference between the groups (3.8% in Pre-VLFF vs. 3.6% in the post-VLFF group, *p* = 0.950; Table 3).

**Table 3:** Radiation exposure, fluoroscopy time, and MACE at 1 year in all patients as well as Pre-VLFF and Post-VLFF subgroups.

**Outcome**	**All (*n* = 326)**	**Pre-VLFF 15 fps (*n* = 133)**	**Post-VLFF 3.8 + 7.5 fps (*n* = 193)**	***P*-value**
Air kerma (mGy)	955 ± 69 528 (270–1135)	1,714 ± 140 1,197 (683–2062)	433 ± 27 302 (206–517)	<0.0001
Contrast use (ml)	116.6 ± 3.8	116.7 ± 6	116.5 ± 4.9	0.700
Fluoroscopy time (mins)	16.06 ± 0.78	17.06 ± 1.29	15.38 ± 0.98	0.529
All cause mortality	7 (2.1)	3 (2.3)	4(2.1)	0.911
TLF	4 (1.2)	2 (1.5)	2 (1)	0.706
MI	2 (0.6)	0 (0)	2 (1)	0.239
Stroke	1 (0.3)	1 (0.8)	0 (0)	0.228
MACE	12 (3.7)	5 (3.8)	7 (3.6)	0.950

*Values are n (%), mean ± SEM, or median (interquartile range)*.

*fps, frames per second; MACE, Major Adverse Cardiac Events; mGy, milligray; MI, Myocardial Infarction; VLFF, Very Low Frame Rate Fluoroscopy Protocol; TLF, Target Lesion Failure*.

The Multivariate analysis found that fluoroscopy time (*P* < 0.001, *B* = 53.63, 95% CI = 47.01 to 60.25) and lower frame rate (*P* < 0.001, B = −1,186.90, 95% CI = −1,336.91 to −1,036.89) were the factors that independently predicted radiation exposure.

## Discussion

This study was conducted as part of a continuous effort being made at our centers to reduce radiation exposure in the catheterisation laboratory. In this study, we have demonstrated that adoption of a simple protocol of reducing fluoroscopy frame rate can decrease the radiation exposure to patient by 74.7%.

Multiple prior studies have demonstrated that decreasing the fluoroscopy frame rate from 15 to 7.5 fps can lead to significant fall in radiation exposure (7, 9, 11, 13, 14). This study shows that frame rates as low as 3.8 fps in combination with 7.5 fps, can be utilized safely during PCI, resulting in an even further reduction in radiation exposure in comparison with previous studies (Table 4). Reduction in frame rates can result in decreased temporal resolution of the image and may also increase the image “jerkiness” (2, 14, 15). However, this has not been shown to compromise the quality of the procedure or affect the procedure related outcomes. Based on these observations, fluoroscopy at 7.5 fps has been considered as non-inferior to fluoroscopy at 15 fps and is being used satisfactorily for lesion assessment and stent placement (7, 14). The formulation of the VLFF protocol is intended to further reduce the radiation exposure without compromising the quality of intervention. Frame rates of 3.8 fps result in further slowing of the image, but we utilized 3.8 fps while performing steps which usually don't require a higher resolution such as advancement of catheters till aortic root, engagement of guide catheter, negotiation of wire across the coronary lesion, tracking of pre-dilation and post-dilation balloons. However, we recommend a transition period for the operator and laboratory staff to familiarize themselves with the image quality associated with VLFF protocol. This can be done by gradual incorporation of fluoroscopy using lower frame rates in daily practice as per the operators' discretion.

**Table 4:** Features of the current study as well as previous studies with similar radiation reduction protocols.

**Study**	**Study population**	**Frame rates**		**Other protocol features**	**Radiation reduction**	**Other results**
		**Standard protocol**	**Radiation reduction protocol**			
Chon et al. (7)	Consecutive PCI procedures excluding emergency and CTO procedures	Fluoro:15 fps Cine:15 fps	Fluoro:7.5 fps Cine:10 fps	(1) Cine was only used for baseline coronary angiography (CAG), subsequent CAG, stent positioning, and final CAG, (2) Selective fluoroscopic image storing was used for balloon inflation, stent insertion, thrombus aspiration, and intravascular ultrasonography	AK-21.22% reduction DAP-19.38% reduction	No statistical difference in:(1) Fluoroscopy time (2)Image quality
Wassef et al. (9)	Both CAG and PCI	Fluoro:15 fps	Fluoro:15 fps cohort, 7.5 fps cohort	(1) Increasing the thickness of x-ray beam spectral filters for acquisition imaging, (2) Reducing the frame rates to 7.5 fps, (3) Reducing detector dose rate in acquisition imaging (4) Setting the default fluoroscopy dose rate mode from normal to lowor a combination of these changes	AK-62% reduction in the 7.5 fps cohort	(1) No statistical difference in Fluoroscopy time. (2) Cine runs-Statistical increase in post protocol CAG patients (3) Increase in mean number of stents in protocol group, but not in 7.5 fps cohort
Abdelaal et al. (13)	Both CAG and PCI	Fluoro:15 fps Cine:15 fps	Fluoro:7.5 fps Cine:15 fps	Operators encouraged to use fluoroscopy store	Operator dose-30% reduction DAP-19% reduction	No statistically significant difference in:(1) Fluoroscopy time (2) Procedural duration (3) Contrast volume
Pyne et al. (14)	Both CAG and PCI	Fluoro:15 fps Cine:15 fps	Fluoro:10 fps Cine:10 fps	–	AK-33% reduction (unadjusted)	No statistically significant difference in:(1) Fluoroscopy time (2) Contrast volume (3) Angiographic quality score
Hansen et al. (11)	Both CAG and PCI	Fluoro:10 fps Cine:15 fps	Fluoro:7.5 fps Cine:15 fps	–	AK-18.35 and 11.66% reduction in PCI and CAG, respectively, DAP-21.02 and 21.61% reduction in PCI and CAG, respectively	(1) No difference in MACE at 30 days and 6 months. (2) No difference in Fluoroscopy time (3) Contrast volume was lower in study population in both CAG and PCI group
Gupta et al. (Present study)	PCI procedures	Fluoro:15 fps Cine:15 fps	Fluoro:3.8 + 7.5 fps Cine:15 fps	(1) Fluoroscopy of 3.8 fps for advancement of guide catheter hooking, coronary artery wiring, tracking the pre-dilation, and post-dilation balloon. (2) Fluoroscopy of 7.5 fps for lesion assessment and stent positioning. (3) Cine use for baseline angiography, assessing procedural complications, and final angioplasty result. (4) Increasing use of fluoroscopy store instead of cine for retrospective review	AK-74.7% reduction	No statistically significant difference in:(1) Fluoroscopy time (2) Contrast volume (3) Procedural complications (4) Procedure success rate (5) MACE at 1 year

This study demonstrated that use of VLFF protocol during PCI was not associated with increase in fluoroscopy time, contrast used, number of stents used or complications during the procedures. This protocol was used successfully even in complex cases like chronic total occlusion (CTOs), bifurcation PCI, primary PCI, left main disease and multivessel disease. Hence, PCI can be performed using VLFF protocol with the same competency as the traditionally utilized 15 fps. Use of the VLFF protocol during PCI was not associated with increase in MACE (all-cause mortality, TLF, MI, and stroke) at 1 year, when compared with 15 fps. This indirectly provides us with the confidence that use of VLFF protocol doesn't lead to procedural complications that may manifest as MACE in the long run.

Radiation exposure can have far reaching consequences for both the patient and operator. Interventional cardiologists and patients, due to their proximity to the X-ray beam, are subjected to increased radiation exposure as compared to other medical staff (16). This exposes them at an increased risk of radiation induced damage. Ionizing radiation has two main types of side effects. Stochastic effects have no threshold and the probability of these is dose dependent. These include carcinogenic and genetic effects with an increased risk of solid tumors (6, 17). Deterministic effects are seen when exposure to radiation exceeds a threshold level beyond which there is a sharp increase in incidence rate. These are also known as tissue reactions and include hair and skin changes, cataracts and cardiovascular disease (18). The accepted threshold for deterministic injury for patients is 2Gy (14).

For the purpose of this study we have used Air Kerma as the primary endpoint which can be used to estimate entrance skin dose and is a good measure of deterministic injury in patients which is more pertinent in acute fluoroscopic radiation as is seen in interventional cardiology (19). Air Kerma is defined as the radiation exposure at a defined point which is at a 15 cm distance from the gantry isocenter toward the X-Ray tube and hence would not be affected by the route of intervention. While our study showed a statistically significant difference in the route of intervention between the two groups with a significantly larger proportion of transradial PCI procedures in the Post-VLFF group, the multivariate analysis showed that air kerma levels were dependent on fluoroscopy time rather than the route of intervention.

Evidence regarding increase in radiation exposure with radial route is still rather inconsistent, with some studies reporting a significant difference in Dose Area Product (DAP) while others have reported no difference (20). However, the RAD-MATRIX study used dosimeters to clearly demonstrate a significant increase in radiation exposure to the operator with the radial approach as compared to femoral approach (21). With the increase in transradial approach in PCI procedures, it is more important than ever to find new strategies for radiation dose reduction due to the potential increase in exposure.

The accepted standard of radiation safety is ALARA (As Low As Reasonably Achievable) (22, 23) and radiation dose optimisation by adopting VLFF protocol is an excellent example of this model. The frame rates at which fluoroscopic images are generated can be changed in modern angiographic systems. This control provides an easy and effective way to reduce the radiation burden on the patient as well as the catheterisation laboratory staff. This is especially important in a high volume center like ours, where as many as 15 cases a day are done including complex procedures. Most importantly VLFF protocol provides the operator a freedom to utilize a combination of frame rates as per their discretion by keeping the frame rate “As Low As Reasonably Achievable” for adequate imaging.

Hence fluoroscopy frame rates as low as 3.8 fps in combination with 7.5 fps can be used for PCI without a corresponding increase in procedural complications and MACE.

### Study Limitations

This was single center, observational study which has its own inherent limitations. Randomization could not be done, however baseline and procedural characteristics were compared to ensure that the two groups were as comparable as possible. The study was powered only to detect the difference in the radiation exposure and was not powered for the secondary outcomes. While there was no apparent difference in patient related outcome, the follow-up period was short and observation over longer periods may be required to draw a more reliable conclusion regarding patient safety. It is also important to note that the significant reduction in radiation exposure was due to the combined effect of lower frame rates as well as reduced cine runs. In addition to this, the operators were possibly more aware of the need for radiation reduction after the implementation of VLFF protocol. This may be have led to undeliberate alterations in technique to enhance exposure reduction thus confounding the results. A multi-center, multi-operator, prospective study is required to validate and further streamline these findings.

### Impact on Daily Practice

To our knowledge, this is the first study utilizing the Very Low frame rate fluoroscopy (VLFF) protocol and demonstrating the feasibility, efficacy and safety of using fluoroscopy frame rates as low as 3.8 fps in combination with 7.5 fps for PCI. Another advantage of this study is the implementation of the protocol even in complex procedures with similar patient outcome. This VLFF protocol significantly reduces radiation exposure to the patient, interventional cardiologist and catheterization laboratory staff. This reduction in radiation exposure may translate into reduction in radiation hazards like cancer, cataracts, non-malignant skin damage, and cardiovascular diseases.

## Conclusion

Use of VLFF protocol during PCI is associated with highly significant reduction in radiation dose exposure without increasing fluoroscopy time and procedural outcome and patient related outcome. This is an effort to constantly upgrade our radiation safety protocols to minimize exposure as much as possible.

## Data Availability Statement

The raw data supporting the conclusions of this article will be made available by the authors, without undue reservation.

## Ethics Statement

The studies involving human participants were reviewed and approved by Institutional Ethics Committee, Base Hospital Delhi Cantt. Written informed consent for participation was not required for this study in accordance with the national legislation and the institutional requirements.

## Author Contributions

AG: conceptualization, data curation, investigation, methodology, supervision, manuscript writing, and review. SC: data curation, data analysis, investigation, manuscript writing, and review. RV: conceptualization, methodology, supervision, manuscript writing, and review. PB: data curation, investigation, manuscript writing, and review. KP and RD: data analysis, manuscript writing, and review. NM, PM, and NS: manuscript writing and review. All authors have contributed significantly to this manuscript.

## Conflict of Interest

The authors declare that the research was conducted in the absence of any commercial or financial relationships that could be construed as a potential conflict of interest.
